# A Quality Improvement Collaborative for Pediatric Sepsis: Lessons Learned

**DOI:** 10.1097/pq9.0000000000000051

**Published:** 2017-12-29

**Authors:** Raina Paul, Elliot Melendez, Beth Wathen, Gitte Larsen, Laura Chapman, Derek S. Wheeler, Toni Wakefield, Charles G. Macias

**Affiliations:** From the * Division of Emergency Medicine, Ann and Robert H. Lurie Children’s Hospital of Chicago, Feinberg School of Medicine Northwestern University, Chicago, Ill.; † Division of Critical Care, Johns Hopkins All Children’s Hospital, St. Petersburg, Fla.; ‡ Children’s Hospital Colorado, Pediatric Intensive Care Unit, Aurora, Colo.; § Primary Children’s Hospital, University of Utah School of Medicine, Salt Lake City, Utah; ¶ Hasbro Children’s Hospital, Alpert Medical School, Providence, R.I.; ‖ Cincinnati Children’s Hospital Medical Center, University of Cincinnati College of Medicine, Cincinnati, Ohio; ** Dell Children’s Medical Center, Austin, Tex.; †† Department of Pediatrics, Section of Emergency Medicine and The Center for Clinical Effectiveness, Baylor College of Medicine/Texas Children’s Hospital, Houston, Tex.; and ‡‡ The Center for Clinical Effectiveness, Baylor College of Medicine/Texas Children’s Hospital, Houston, Tex.

## Abstract

**Background:**

Sepsis is a leading cause of morbidity and mortality in children worldwide. Barriers exist for timely recognition and management in emergency care settings. This 1-year quality improvement collaborative sought to reduce mortality from sepsis.

**Methods:**

Fifteen hospitals participated initially. We included children with a spectrum of illness from sepsis to septic shock. The intervention bundle focused on recognition, escalation of care, and the first hour of resuscitation. We conducted monthly learning sessions and disseminated data reports of site-specific and aggregated metrics to drive rapid cycle improvement.

**Results:**

Seven sites contributed enough data to be analyzed. Of the 1,173 pediatric patients in the total cohort, 506 presented with severe sepsis/septic shock. Quarterly data demonstrated a mean improvement in initial clinical assessment from 46% to 60% (*P* < 0.001) and in adherence to the administration of first fluid bolus within 15 minutes from 38% to 46% (*P* < 0.015). There was no statistically significant improvement in other process metrics. There was no statistically significant improvement in mortality for the total cohort (sepsis to septic shock) or either of the subgroups in either 3- or 30-day mortality.

**Conclusions:**

A quality improvement collaborative focused on improving timely recognition and management of pediatric sepsis to septic shock led to some process improvements but did not show improvement in mortality. Future national efforts should standardize definitions and processes of care for sepsis to septic shock, including the identification of a “time zero” for measuring the timeliness of treatment.

## INTRODUCTION

### Background

Severe sepsis is one of the leading killers of children worldwide, accounting for over 8 million deaths annually.^1^ From 2004 to 2012, pediatric severe sepsis prevalence in the United States increased (3.7–4.4%), with an associated 176,000 hospitalizations and mortality of 8.2% (11,000 deaths) in 2012.^3^ However, the prevalence of sepsis, severe sepsis, and septic shock in epidemiology studies vary based on definitions used. Furthermore, mortality statistics do not account for variability in recognition and delivery of care.^2–7^

### Common Local Problem

Studies have shown that early treatment of septic shock improves outcomes.^8–19^ Paul et al. demonstrated that when adjusting for severity of illness at emergency department (ED) presentation, patients who received 60 mL/kg of isotonic intravenous (IV) fluid within 60 minutes had a 57% shorter hospital length of stay (LOS), and a 42% shorter Pediatric Intensive Care Unit (PICU) LOS. They associated adherence to a 5-step bundle with a nearly 60% reduction in hospital and PICU LOS.^15^

Barriers to delivering timely sepsis care exist.^16^ Barriers in tertiary pediatric ED settings resulted in poor adherence to the rapid administration of IV fluids, vasoactive agents, and antibiotics.^12,13,15^ A failure to recognize the signs and symptoms of septic shock, especially signs of impaired tissue perfusion and hypotension, leads to delayed fluid resuscitation and antibiotic therapy. These delays contribute to worse outcomes in children.^17,18^ As there are no specific blood tests or diagnostic tools to diagnose sepsis rapidly, heightened awareness of the constellation of signs and symptoms associated with sepsis is critical.

In a survey of emergency room physicians, Thompson and Macias^20^ identified a wide variability in provider perceptions and institutional initiatives for identification and management of sepsis, severe sepsis, and septic shock. To address this variability in pediatric sepsis management, we initiated a multi-institution rapid cycle improvement collaborative. The intent of the collaborative was for practice-based teams to learn from one another, test changes, and use their collective experience and data to improve recognition and treatment and decrease mortality and morbidity, for children with septic shock.

### Specific Aims

The aim of this collaborative was to reduce 3-day and 30-day mortality due to sepsis over a 1-year period. The collaborative also aimed to achieve 95% compliance with the following key sepsis diagnosis and management processes: initial clinical assessment, fluid bolus administration, and antibiotic administration.

## METHODS

### Context and Setting

The pediatric sepsis collaborative was a joint effort of the Children’s Hospital Association (CHA) and the Pediatric Septic Shock Collaborative. CHA consists of 225 member hospitals that embody 90% of all children’s hospitals in the United States. The Pediatric Septic Shock Collaborative is an initiative of the Section of Emergency Medicine of the American Academy of Pediatrics.

### Collaborative Structure

**Fig. 1.**
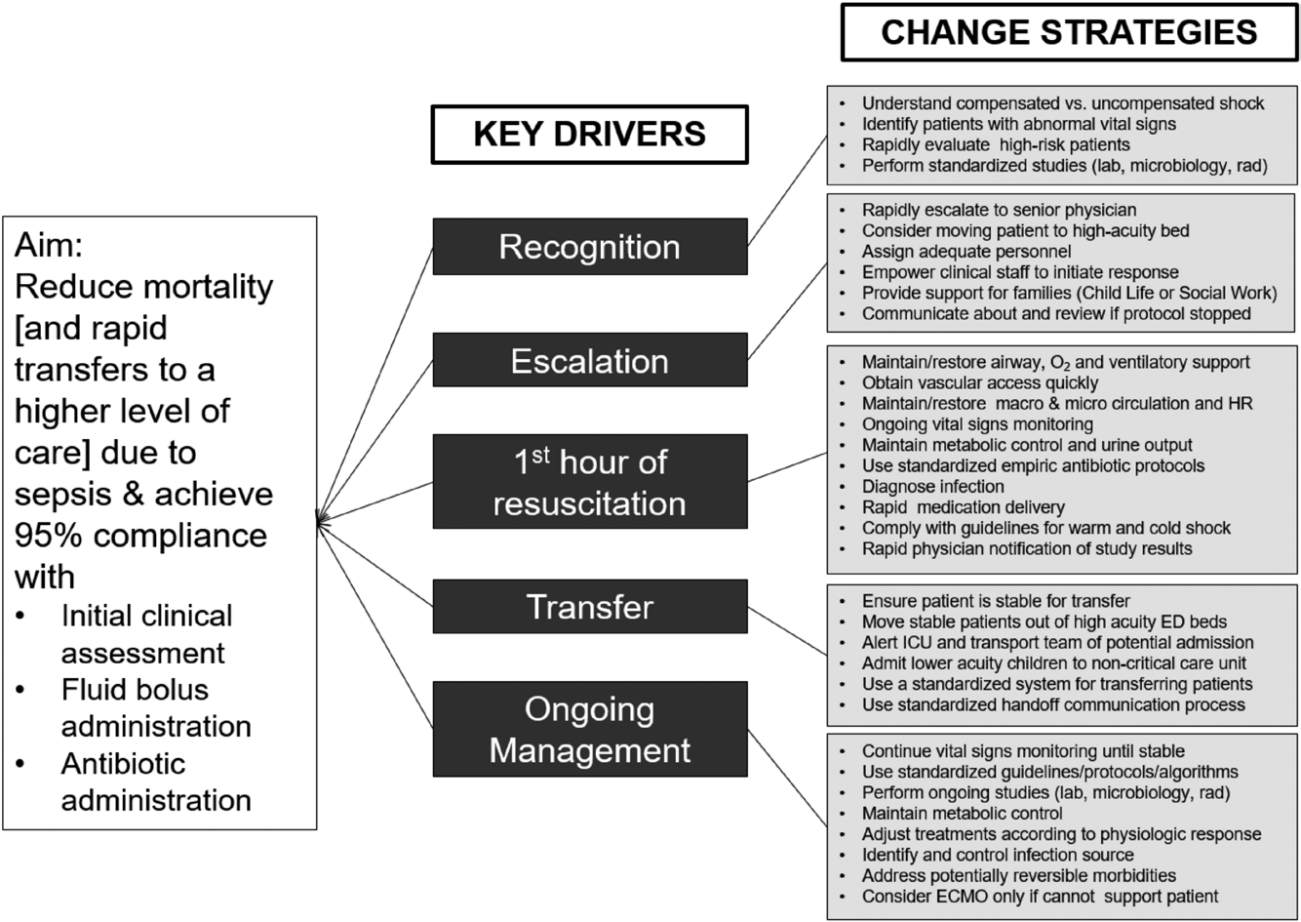
Children’s Hospital Association sepsis key driver diagram.

In early 2012, we established an Expert Advisory Panel of relevant stakeholders. The panel included pediatric emergency medicine, critical care, and infectious disease physicians, pediatric emergency medicine and critical care nurses, and clinical pharmacists. The advisory panel convened by a series of webinars and 1 in-person meeting to develop a charter, a comprehensive change package including key driver diagram, and specific process and outcome measures. The key drivers included early recognition, escalation of care, the first hour of resuscitation, patient transfer, and ongoing management until patient stabilization (Fig. 1). The project targeted pediatric sepsis management in the ED, medical/surgical units, and intensive care units (ICUs).

The collaborative utilized the Institute for Healthcare Improvement’s Model for Improvement, incorporating a dynamic process of small tests of change using the Plan-Do-Study-Act methodology.^21^ Four virtual learning sessions (quarterly) and monthly webinars allowed participating hospitals to share successes and barriers and provided a forum for brainstorming ideas. In addition to sharing ideas between teams, the quarterly Learning Sessions also provided QI tools including communication strategies, screening tools, staff education and training, reliability science, Pareto principles to analyze/address common barriers, data reporting methods, sepsis cohort definitions, run chart rules, and data quality considerations. Each institution adapted these tools for local infrastructures and resources. The 1-year action period began in June 2012.

### Collaborative Membership

The collaborative was open to all CHA member hospitals. Fifteen hospitals agreed to participate. The enrolled hospitals varied in size, demographics, patient severity of illness, and infrastructure (Table 1). Each participating site selected their multidisciplinary team members and senior leaders; they each received an Improvement Handbook that provided an overview of the collaborative process, and an Instruction Manual outlined specific process steps.

**Table 1.**
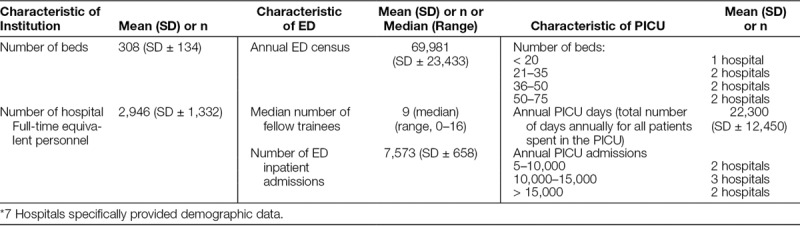
2012–2013 Demographic Data of Participating Hospitals*

### Patient Population

This collaborative used the Pediatric Advanced Life Support septic shock guidelines from the American Heart Association in additional to other national guidelines.^20–25^ Based on the 2005 International Pediatric Sepsis Consensus Conference definitions, patients with sepsis, severe sepsis, and septic shock were recommended for inclusion.^26^ Institutions could include septic patients with less severe disease, but all sites were encouraged to include all patients who met criteria for severe sepsis or septic shock.^26^ Sites were strongly advised to adhere to these definitions, but ultimately, each participating site reported patient data per their institutional convention.

Each hospital selected their target populations. The majority of the collaborative focused on interventions in the ED. Some sites targeted other areas including Medical/Surgical units, Hematology/Oncology/Bone Transplant units, and Critical Care Units (Cardiac critical care, Cardio-thoracic ICU, and PICU). However, we excluded patients in the Neonatal ICU and outpatient clinics.

### Sepsis Improvement Bundles

Several investigators have developed algorithms and intervention bundles for children and neonates to enhance timely recognition and response to septic shock.^19–23^ For this collaborative, most change efforts focused on 3 areas: (1) recognition; (2) escalation of care; and (3) the first hour of resuscitation. The project also addressed subsequent transfer to an ICU if admitted to a general pediatric ward as well as ongoing management. Each participating site received strategies for improvement that included ways to learn from existing processes and creatively overcome barriers, create processes and structures to support improvements, and use incentives to maintain engagement of staff and stakeholders.

### Study of the Intervention

We recorded baseline measurements of outcome measures for a 1-year period (7/2011–6/2012) before initiation of the collaborative. Baseline data for all other measures were collected from the 3 months before project initiation when available (4/2012–6/2012), or from the first 3 months the institution participated in the collaborative.

Although the collaborative measures were based largely on the Surviving Sepsis Campaign^27^ and the Pediatric Advanced Life Support guidelines, institutions varied in the manner in which they gathered data. We defined compliance with process measures as the proportion of patients for whom time goals were met among the population of septic patients who required each intervention.

Each institution defined their start time (time zero). Their choices included any of the following: time of ED arrival, first abnormal vital signs, clinician determination and notation of meeting sepsis criteria, sepsis order set initiation, initiation of a best practice alert, or presence of an elevated pediatric early warning system (PEWS) score. Time zero as operationalized by each institution remained constant throughout the study period.

Barriers to delivery of ideal care were assessed each month using a template form. Individual sites reported perceived and measured barriers as they relate to implementation of process metrics, cultural factors, and resource limitations. Additionally, these barriers were expounded upon during the quarterly webinar presentations by each site.

### Measures

#### Outcome Measures.

Primary outcomes included 3- and 30-day mortality rates for patients with sepsis, severe sepsis, or septic shock.

#### Process Measures

1) Timeliness of initial assessment: defined as assessment occurring within 20 minutes of arrival in the ED, ICU, or inpatient unit. The initial assessment required the acquisition of a full set of vital signs necessary for early recognition of sepsis.
2) Timeliness of fluid resuscitation:
  a) Initial 20 ml/kg isotonic IV fluid bolus: administration within 15 minutes from time zero
  b) 60 ml/kg of isotonic IV fluid: administration within 60 minutes from time zero if 60 ml/kg were ordered
3) Timeliness of antibiotic administration: administration within 60 minutes from time zero

#### Balancing Measures.

False positive rate: defined as all patients who did not ultimately have sepsis as a diagnosis out of all patients who initially met screening criteria for septic shock.

Except for mortality data, participating sites reported process, outcome, and balancing measures monthly. They reported mortality data quarterly. Each institution also submitted a narrative report detailing their self-assessment of progress, barriers, and breakthroughs as well as future steps. Monthly reports included run charts of all measures on an institutional and aggregate level, as well as customized written feedback from collaborative leadership.

#### Analysis.

For analysis, we only included data from hospitals that contributed > 90% of the required data. All hospitals included in the analysis had at least 1 month of data reported in the final quarter. Each site contributed data for sepsis, severe sepsis, septic shock or all 3 as their resource capability allowed. Thus, some sites contributed data for sepsis patients but not severe sepsis or septic shock patients, and vice versa. For mortality calculations, we excluded hospitals that were unable to provide a year of baseline data.

We report demographic characteristics as medians, with interquartile ranges, and analyze categorical data by chi-square analysis. Comparisons between baseline and postbaseline values for individual hospitals were made using Fisher’s exact tests. Aggregate comparisons between baseline and postbaseline values were made using chi-square analysis. We assessed normality of the data before using parametric statistical tests and established a *P* < 0.05 for statistical significance. We used SAS version 14 for Windows (Cary, N.C.) for statistical analysis. QI charts (Process Improvement Products, Austin, Tex.) were used for the statistical process control analyses. We used time series analysis (run charts and statistical process control charts) to report mortality and process compliance metrics (time to first and third bolus, and time to antibiotics).^28^

Hospitals with missing baseline data used the first quarter of data for the collaborative as their baseline data. For hospitals with missing data, we imputed data using the Last Observation Carried Forward or Last Observation Carried Backward methods (https://communities.sas.com/t5/SAS-Procedures/missing-data-imputed-using-LOCF/td-p/99781).

#### Ethical Issues/Institutional Review Board/Data Use Agreements.

Each team submitted a local institutional review board application if required by their site and submitted a standardized Data Use Agreement to CHA.

## RESULTS

Seven hospitals were able to submit data for all measures across the entire study period, and therefore, were included in the analysis. We detail the demographics of analyzed hospitals in Table 1. The mean number of hospital beds was 308 (SD ± 134). The mean annual ED census was 69,981 (SD ± 23,433). The mean PICU number of days was 22,330 (SD ± 12,450), which represents the total number of days annually for all patients spent in the PICU. Ninety-three percentage of the analyzed team members participated in all learning sessions; 66% attended the didactic webinars.

Of these hospitals, some contributed data for sepsis overall, some for severe sepsis/septic shock, and some for both (Table 2). The 7 included-institutions identified total sepsis patients by the following methods: manual identification from International Classification of Diseases (ICU) and step-down units, applying Goldstein definitions, ICD-9 codes consistent with sepsis, severe sepsis, and septic shock or the firing of best practice alerts or use of order sets. For identification of severe sepsis and septic shock patients, all analyzed sites relied on the use of 2 sepsis ICD-9 codes (severe sepsis, 995.92 and septic shock, 785.52) or manual identification using Goldstein definitions.

**Table 2.**
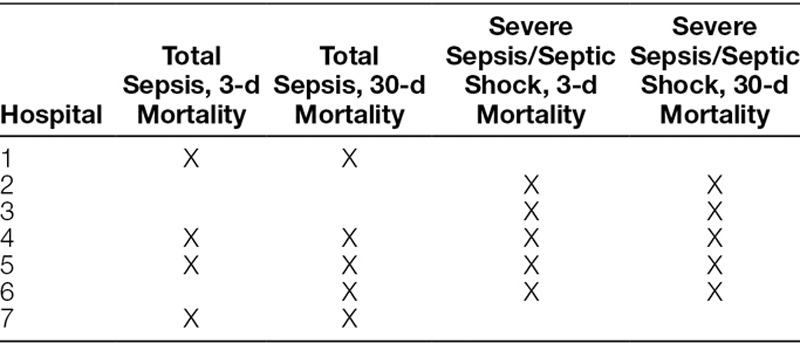
Institutions That Contributed Complete Data Throughout the Collaborative Cycle

During the study period, 1,173 pediatric patients with suspected sepsis, severe sepsis, or septic shock were treated (619 baseline, 554 postintervention). Five hundred six were reported to have severe sepsis or septic shock (275 baseline, 231 postintervention). The overall 30-day mortality rate for total sepsis patients (i.e., all categories: sepsis, severe sepsis, and septic shock) was 4.2%. The overall 30-day mortality rate for those reported as having severe sepsis and septic shock was 7.9%.

### Process Measures

**Fig. 2.**
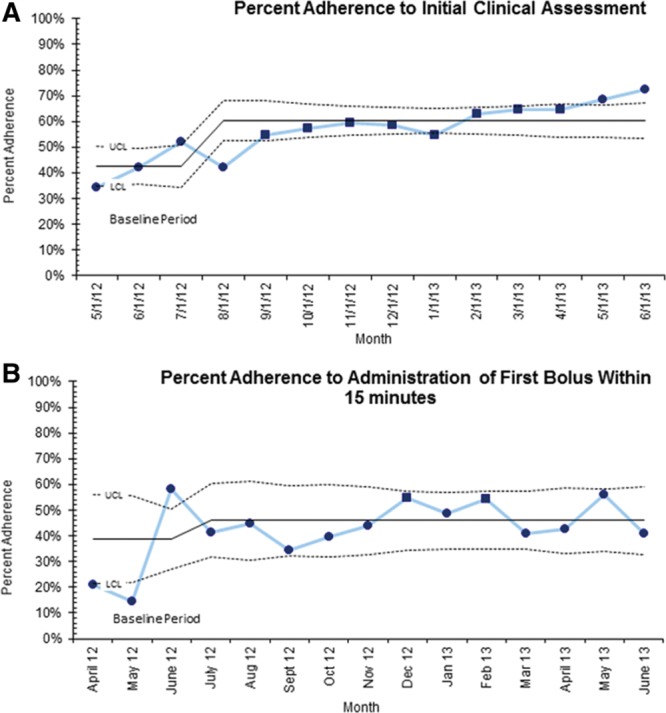
Process measures. A, Percent adherence to initial clinical assessment within 20 minutes, statistical process control chart (P chart) ▪ denotes 8 points above initial baseline justifying centerline shift demonstrating special cause. B, Percent adherence to administration of first fluid bolus within 15 minutes, statistical process control chart (P Chart) ▪ denotes 2 points in the outer third of the chart among 3 consecutive points, representing special cause.

Quarterly data demonstrated mean improvement in initial clinical assessment from 46% to 60% (*P* < 0.001) and in adherence to the administration of first fluid bolus from time zero from 38% to 46% (*P* < 0.015). Figures 2A, B display the monthly data demonstrating variation in these processes over time. There was no statistically significant improvement in percentage adherence to the remainder of the process measures including administration of 3 boluses within 1 hour (50–57%) and administration of antibiotics within 1 hour (56–59%).

### Outcome Measures

**Fig. 3.**
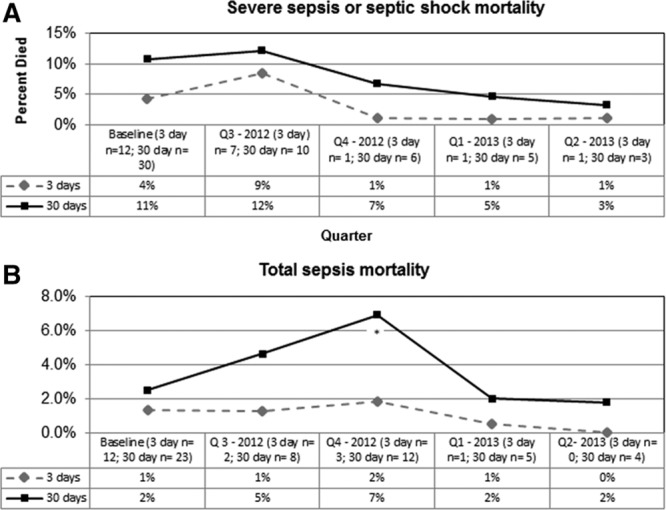
Outcome measures. A, Three- and 30-day mortality for severe sepsis and septic shock (percentages calculated using denominators reported for each quarter). *Statistically significant, *P* < 0.05. B, Three- and 30-day mortality for total sepsis patients (percentages calculated using denominators reported for each quarter). *Statistically significant, *P* < 0.05.

For the 7 hospitals that contributed full data across the study period, there was no statistically significant reduction in 3- or 30- day mortality for patients with severe sepsis and septic shock when comparing baseline data to the final quarter of the collaborative (Fig. 3A). Similarly, the 3- and 30-day mortality for patients with total sepsis were unchanged (Fig. 3B). It is important to note, as detailed in Table 2, the hospitals that reported data for the total sepsis cohort were not congruent with the hospitals that reported data for the severe sepsis/septic shock cohort.

### Balancing Measure

There was not enough data submitted on the recommended balancing measure to analyze.

## DISCUSSION

In this study, we did not identify any reduction in our primary outcomes of 3- and 30-day mortality from sepsis, severe sepsis, or septic shock during the collaborative period. Improvement in process measures such as timely initial assessment and initial fluid bolus were observed, but the timeliness of antibiotic administration did not improve. Figure 2A demonstrates 8 points above initial baseline justifying centerline shift and demonstrating special cause. Likewise, Figure 2B shows special cause by 2 of 3 consecutive points in the outer one-third of the control limit. However, this evidence of improvement was not sustained.

Improving recognition of the early signs of septic shock (ie, time to first clinical assessment) is a critical component in improving outcomes. This measure did show improvement over time. Although specific interventions to reduce the time to initial clinical assessment varied among institutions, all sites emphasized education around early recognition. Several organizations incorporated decision support in the electronic medical record or additional triage screening that triggered an alert based on the patient’s risk criteria and initial clinical assessment.

The collaborative also provided an opportunity to improve organizational processes to decrease delays in care delivery through protocol-driven care. Institutions were at various phases of improvement work before the collaborative start. However, only 2 of the included hospitals had established robust sepsis protocols within standard workflows. By study end, all data contributing hospitals had developed a sepsis protocol, implemented an order set, conducted stakeholder barrier analysis, and executed widespread education.

Teams identified several local care barriers. Delays in antibiotic administration resulted from pharmacy processing and delivery barriers. This delay may be due to the remote location of the pharmacy from the ED at most sites. Delivery of 60 ml/kg of Intravenous Fluids (IVF) for those with severe sepsis and septic shock was also problematic across all sites. Reported barriers included lack of understanding regarding which patients need aggressive resuscitation, hesitance to use a small gauge IV for fluid delivery, and concerns regarding bedside nursing resources for rapid infusion methods. Interestingly, a standardized order set (present in all analyzed sites) was not an intervention that improved fluid delivery, given the reported barriers.

Three adult trials with strict adherence to an early goal-directed therapy septic shock bundle focused on the 6-hour time window after initial recognition and fluid resuscitation. These trials failed to show significant improvements.^29–31^ Rather than endorsing early goal-directed therapy or laboratory-based criteria for diagnosis and management of sepsis, this project focused on improving timely recognition of sepsis and escalation to more aggressive management. Participation in the collaborative itself likely contributed to the heightened local awareness of sepsis as a diagnosis. This increased awareness may have increased the coding of less severely ill patients with a lower risk of mortality. However, our reported overall mortality rate for the total sepsis and severe sepsis populations of 4.2% and 7.9%, respectively, are within the range of reported epidemiologic estimates.^2^

### Lessons Learned

The waning participation rate in this collaborative highlights an opportunity for future collaboratives focusing on pediatric sepsis. Three hospitals dropped out of the collaborative early in the process citing difficulties with collecting data. Several others did not contribute enough data to be analyzed for the entire period and all sepsis cohorts. Queries of the team leaders from these institutions uniformly noted insufficient support and resources for identifying the correct cohort as well as reporting the required variables.

Unlike many other pediatric diagnoses, sepsis is an extremely heterogeneous entity resulting in idiosyncrasies that can be onerous for easy data query. Use of administrative data (ICD-9 and 10 codes) for data capture is problematic as providers may inconsistently code for sepsis.^3^ The collaborative strongly recommended not relying on coding alone for cohort identification. Sites included in the analysis reported identification using an individualized combination of several methods described previously. These institutions also reported resorting to manual chart review for accurate capture of sepsis patients and characterization of each encounter. Sites that could not contribute data noted that these methodologies were too resource intensive.

Additionally, teams reported difficulties with standardizing time zero for the development of sepsis. This lack of uniformity for time zero created significant issues for data collection and analysis. The final 5 sites that were analyzed, however, all reported a consistent time zero throughout the study period. Future collaborative efforts should aid institutions with explicit methods for engaging administrative leadership to increase support for these efforts as well as creating uniform definitions a priori.

The 2 highest performing sites were lead by passionate physician champions. These 2 sites reported improvement in all process metrics and 1 site reported improvement in mortality as well. They also noted frequent local project meetings; uniform understanding and definitions defined a priori; clinician acceptance of existing evidence; robust educational efforts and standardized workflows across care settings for sepsis patients. These 2 sites also reported frequent personal and aggregate feedback regarding metric performance as a means of sustaining team engagement. These 2 sites also utilized second order high-reliability strategies, such as utilization of a shock clock, prechecked order sets and explicit recommendation to use a pressure bag for fluid delivery. We recommend inclusion of these components for future sepsis QI efforts.^32^

### Limitations and Interpretation

Our study presents several limitations. A primary goal of this collaborative was to focus on rapid cycle institutional change, rather than standardizing care among all institutions. As such, definitions of sepsis and determination of time zero varied between institutions. Because the collaborative focused on rapid cycle institutional change, emphasis was placed upon what was locally feasible. Although Goldstein et al.^26^ present specific definitions for sepsis, severe sepsis, and septic shock, these are difficult to apply prospectively and thus some institutions utilized clinician judgment for inclusion. However, the analyzed institutions utilized Goldstein definitions and 2 specific ICD-9 codes for the subset of more critical patients with severe sepsis and septic shock.

A second limitation involves a likely increased recognition of sepsis patients throughout the course of the collaborative. As sites became more aware of the condition, the reported incidence of sepsis may have increased and diluted the denominator with less sick patients. However, the numbers of patients in the pre- and postintervention groups are similar for both severe sepsis and septic shock cohorts. These results may indicate that an over-coding bias was not present in our final analyzed cohort.

Third, the 2 sites that had already begun robust QI interventions before collaborative initiation likely contributed to the improvements seen in the collaborative as a whole. However, other institutions also showed local gains for several measures.

## CONCLUDING SUMMARY

This QI collaborative demonstrated improvements in some process measures over time but did not demonstrate a decrease in mortality across the collaborative. Our experience suggests that future national efforts should standardize definitions and processes of care for sepsis as much as possible, including the identification of a “time zero” for measuring the timeliness of treatment. Local institutions should prioritize resources for robust data capture of this heterogeneous entity. Economies of scale could be gained through national standardized and shared platforms as a framework for educating providers at all levels and of all types. Finally, electronic medical record–based clinical decision support tools may drive more timely recognition and more effective management.

## ACKNOWLEDGEMENTS

Collaborators: Advisory group members: Thomas Abramo, MD, FAAP, FACEP, Monroe Carell Jr. Children’s Hospital at Vanderbilt; Sherman Alter, MD, Dayton Children’s Hospital; Jean Christopher, RN, MSN, CNS, WCC, Akron Children’s Hospital; Jim Fortenberry, MD, MCCM, FAAP, Children’s Healthcare of Atlanta; Rainer Gedeit, MD, Children’s Hospital of Wisconsin; Mindi Johnson, RN, MSN, CPN, Children’s Hospital of Michigan; Phuong Lieu, PharmD, Children’s Hospital Los Angeles; Joe Luria, MD, Cincinnati Children’s Hospital Medical Center; Charles Macias, MD, MPH, Texas Children’s Hospital; Binita Patel, MD, Texas Children’s Hospital; Teresa Rushing, Pharm D, BCPS, Children’s Hospital Los Angeles; Beth Wathen, MSN, PMP, CCRN, Children’s Hospital Colorado; Elizabeth (Liz) Wuestner, MSN, RN, Texas Children’s Hospital; Derek Wheeler, MD, FAAP, FCCP, FCCM, Cincinnati Children’s Hospital Medical Center; Eric Williams, MD, MS, MMM, Texas Children’s Hospital. Assistance with the study: Tina Logsdon, Children’s Hospital Association; Cary Thurm, Children’s Hospital Association, Krystle Bartley, Baylor College of Medicine and Texas Children’s Center for Clinical Effectiveness.

## DISCLOSURE

The authors have no financial interest to declare in relation to the content of this article.
